# "Antiseptic" and "Aseptic" Surgery

**Published:** 1891-12-12

**Authors:** 


### SURGERY.

### "ANTISEPTIC" AND "ASEPTIC" SURGERY.

What percentage of the intelligent and educated public has any idea as to the real distinction between "antiseptic" and "aseptic" treatment of wounds? And yet an important and fundamental difference in principle underlies the two processes. The frontiers of the two systems are clearly defined, and the relative values of the two methods at this moment are occupying the minds of some of the greatest surgeons of the day.

"Antiseptic" treatment of one sort or another has been the faithful servant of successful surgeons, whether quacks or otherwise, from time immemorial, but its scientific and universal application we owe to Sir Joseph Lister, and the logical and inevitable outcome of our better understanding of the principles involved has been the evolution of a new surgery —a surgery of "asepsis." Antiseptic methods presuppose the existence of noxious micro-organisms, either in the wound itself or in the media with which the wound may come in contact, and its object is to kill the infecting enemy. Where septic organisms are known to exist, as in a putrid abscess, antiseptic treatment is alone of avail. "Aseptic" treatment would be impossible. Again, suppose a man walking about with bare feet cut his foot with a broken bottle, it is perfectly safe to conclude that the glass is contaminated with micro-organisms, and that they have gained access to the wound. "Antiseptic" treatment here again is the only scientific treatment, and the wound should be washed thoroughly with perchloride of mercury or carbolic acid to ensure the death of the organism and the cessation of its infecting processes. It is to accidents of this kind, where portions of earth find their way into wounds that tetanus, or "lock jaw" as it is popularly called, may ensue, for ordinary garden soil is known to swarm with the bacillus of tetanus, and under certain circumstances, especially where the tissues have been greatly bruised, lock-jaw almost inevitably follows the introduction of such soil into a deep wound. Just as bacteria swarm in the earth we walk on so do they swarm in the air we breathe, the dressings we apply, and the surgical instruments we handle. It therefore appears at first sight that any attempts on the part of the surgeon to grapple with this invisible enemy must be doomed to failure. But this is not so. The antiseptic surgeon restrains their infecting processes by killing the organisms themselves by means of the rigid use of "antiseptics," and the "aseptic" surgeon excludes their presence altogether. We cannot here enter upon the subject of antiseptic surgery, for no one can have crossed the threshold of a well-organised hospital without being made acquainted with the general methods employed to secure "antisepsis" by means of "antiseptic" lotions and antiseptic dressings. The methods of aseptic surgery are, however, known to few in England, and practised by still fewer, so that a detailed account may not be out of place. In the first place, it is the chief care of the surgeon that the dressings he applies and the instruments and water that he uses shall be germ free; that is to say, free of living germs, and the germs must be killed by other means than antiseptic substances, for he is as anxious to exclude from the wound the irritating properties of chemical antiseptics as the germs themselves. The agent he employs to destroy the micro-organisms is heat. Metal instruments, which will not be damaged, are placed in an oven and heated to about 200° Centigrade. This will ensure the death of all living creatures. But it is obvious that the fine temper of cutting instruments would be destroyed at such a temperature, and dressings and ligatures would be burned up or singed; therefore for these another method must be employed. Steam is used for this purpose. Dressings and instruments are placed in a large metal vessel containing water at the bottom, and a large gas flame, to keep the water boiling, is placed underneath; but, inasmuch as water boils at 100°C., any bacteria that can survive such a temperature will remain undestroyed. As a matter of fact, all fully-developed bacteria are killed at such a temperature; nevertheless, the spores can easily survive. The spores may be described as bacteria which have retired into their shells; have, in fact, gone into winter quarters when the surroundings are unfavourable, as, for instance, when there is a scarcity of food, and thus they remain quiescent—as far as we know they remain thus for centuries—until the surroundings are again favourable, food is plentiful, and the temperature to their liking. The difficulty, then, is to destroy these spores, which are quite as numerous as the bacteria and exist everywhere and in everything; on the instruments and on the dressings, as elsewhere. Now these spores are not killed by steam at 100°C., but most of them perish at a somewhat higher temperature. This can be brought about by making the water boil at, say, 150°C., by increasing the pressure by some such means as is employed in a Papin's digester. If apparatus of this kind is not to hand, another method may be employed, namely, that of "fractional sterilization." In this case the materials to be sterilized are subjected to the temperature of steam under ordinary conditions; by this means all the bacteria are killed. Then the materials are allowed to cool down, and the spores develope into mature individuals as soon as the most favourable temperature for their growth has been reached. These mature bacteria are then killed in their turn by again heating up to 100°C. The process must be repeated three times to ensure the conversion of all spores into bacteria and their subsequent destruction by steam. The whole process takes about three days, and is much more expensive and troublesome than the single treatment of dressings at 150°C. under pressure as described above.

In "aseptic" surgery only materials thus sterilized are allowed to come in contact with the wound. The hands of the surgeon are rendered "aseptic" by carefully washing, first with antiseptics, and then with sterilized water. The wound is washed with sterilized water, or sterilized water which contains about a-half per cent. of common salt, as this is found even less irritating than plain sterilized water, the wound is sewn up with ligatures similarly treated, and sterilized dressings are applied. The above is a bare outline of aseptic surgery, and the method fulfils its end, inasmuch as "nothing succeeds like success," and unprecedented success has followed its use by Continental surgeons. That any method can be absolutely "aseptic" is improbable, since, as has been shown, bacteria exist in the air, and may fall upon the surface of the wound and there thrive; but if the tissues are not devitalized by the use of antiseptics they can easily cope with the attacks of single organisms, and their presence in the air of operating-rooms may be reduced to a minimum by the absence of dust either by filtration through cotton wool, as is done in the case of the air entering the Houses of Parliament, or by its removal from the floor and walls by the industrious hand of the scrubber. "Aseptic" surgery implies absolute cleanliness, and the real meaning of this word is alone known to bacteriologits. No surgeon can possibly take up aseptic surgery with any hope of success unless he has learned to approach the meaning, not of relative cleanliness, as understood by the Dutch house frau, but of absolute cleanliness as understood in the bacteriological laboratory.

Antiseptic surgery fails if the smallest number of germs escape the vigilant watchfulness of the surgeon; the tissues themselves are enfeebled by the application of irritating antiseptics, and can no longer exercise their natural antiseptic powers. "Aseptic" surgery encourages this natural power of the tissues, so that if any germs escape the surgeon's hand, they fall into the unrelenting hand of the still vigorous tissues.

